# Methyl 4-methyl­sulfonyl-2-nitro­benzoate

**DOI:** 10.1107/S1600536810021914

**Published:** 2010-06-16

**Authors:** Yan-Jun Hou, Wen-Yi Chu, Jun Sui, Zhi-Zhong Sun

**Affiliations:** a College of Chemistry and Materials Science, Heilongjiang University, Harbin 150080, People’s Republic of China

## Abstract

The title compound, C_9_H_9_NO_6_S, was prepared by the reaction of methanol and thionyl chloride with 4-methyl­sulfonyl-2-nitro­benzoic acid under mild conditions. The dihedral angle between the nitro group and benzene ring is 21.33 (19)° and that between the carboxyl­ate group and the benzene ring is 72.09 (17)°. The crystal structure is stabilized by weak inter­molecular bifurcated C—H⋯O inter­actions occurring in the (100) plane.

## Related literature

For general background to the synthesis and properties of 4-methyl­sulfonyl-2-nitro-benzoic acid methyl ester, see: Carter *et al.* (1991 ▶). For the biological activity of 4-methyl­sulfonyl-2-nitro-benzoic acid methyl ester derivatives, see: Kopsell *et al.* (2009 ▶).
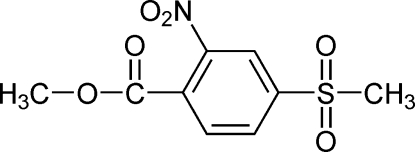


## Experimental

### 

#### Crystal data

C_9_H_9_NO_6_S
*M* *_r_* = 259.23
Monoclinic, 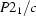

*a* = 9.0108 (12) Å
*b* = 8.7671 (11) Å
*c* = 14.4761 (19) Å
β = 98.955 (2)°
*V* = 1129.7 (3) Å^3^
*Z* = 4
Mo *K*α radiation
μ = 0.30 mm^−1^
*T* = 273 K
0.20 × 0.20 × 0.18 mm

#### Data collection

Bruker SMART APEXII CCD detector diffractometer
Absorption correction: multi-scan (*SADABS*; Sheldrick, 1996 ▶) *T*_min_ = 0.942, *T*_max_ = 0.948
9661 measured reflections
2783 independent reflections
2042 reflections with *I* > 2σ(*I*)
*R*_int_ = 0.028

#### Refinement

*R*[*F* ^2^ > 2σ(*F* ^2^)] = 0.041
*wR*(*F* ^2^) = 0.111
*S* = 1.05
2783 reflections
156 parameters
H-atom parameters constrained
Δρ_max_ = 0.22 e Å^−3^
Δρ_min_ = −0.33 e Å^−3^

### 

Data collection: *APEX2* (Bruker, 2004 ▶); cell refinement: *SAINT* (Bruker, 2004 ▶); data reduction: *SAINT*; program(s) used to solve structure: *SHELXS97* (Sheldrick, 2008 ▶); program(s) used to refine structure: *SHELXL97* (Sheldrick, 2008 ▶); molecular graphics: *SHELXTL* (Sheldrick, 2008 ▶); software used to prepare material for publication: *publCIF* (Westrip, 2010 ▶).

## Supplementary Material

Crystal structure: contains datablocks I, global. DOI: 10.1107/S1600536810021914/kj2147sup1.cif

Structure factors: contains datablocks I. DOI: 10.1107/S1600536810021914/kj2147Isup2.hkl

Additional supplementary materials:  crystallographic information; 3D view; checkCIF report

## Figures and Tables

**Table 1:** Hydrogen-bond geometry (Å, °)

*D*—H⋯*A*	*D*—H	H⋯*A*	*D*⋯*A*	*D*—H⋯*A*
C6—H6⋯O2^i^	0.93	2.54	3.370 (2)	148
C6—H6⋯O3^ii^	0.93	2.59	3.216 (2)	125

Symmetry codes: (i) 
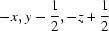
; (ii) 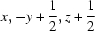.

**Fig. 1.**
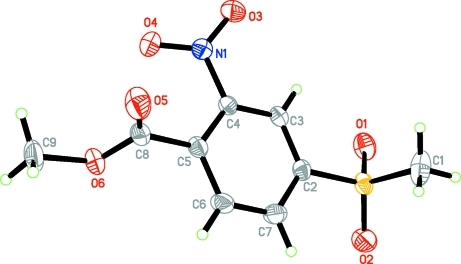
The molecular structure of the title compound, with displacement ellipsoids drawn at the 50% probability level.

**Fig. 2.**
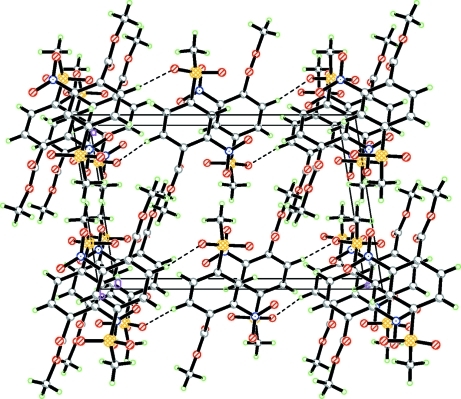
Part of packing of the crystal structure of the title compound, viewed down the b direction. Dashed lines indicate hydrogen bonds.

## supplementary crystallographic information

### Comment

4-methylsulfonyl-2-nitro-benzoic acid methyl ester is used for preparation of mesotrione, which inhibits a critical enzyme, phytoene desaturase, in plant carotenoidbiosynthesis (Kopsell *et al.*, 2009).

The structure of the title compound is shown in Fig. 1. The dihedral angle between the nitro group and benzene ring is 21.33 (19)°. The dihedral angle between the carboxyl group and benzene ring is 72.09 (17)°. The crystal structure is stabilized by weak intermolecular bifurcated C—H···O interactions (the sum of the angles involving H6 as the central atom is 360 (3)°) occurring in the (100) plane (Table 1), resulting in a two-dimensional network (Fig. 2).

### Experimental

Thionyl chloride (250 mmol) was added to a solution of 4-methylsulfonyl-2-nitro-benzoic acid (50 mmol) in anhydrous toluene (250 ml). After stirring the reaction mixture for 10 h at room temperature, the solvent was removed and methanol (100 ml) was added. The reaction mixture was further stirred for 3 h at 323 K. The resulting oil was washed with water (100 ml). After separation from the water phase, the product was concentrated under reduced pressure and the residue was recrystallized from methanol to give the title compound in a yield of 80% (Carter *et al.*, 1991). Crystals suitable for single-crystal X-ray diffraction were obtained by recrystallization from ethanol at room temperature in a yield of 60%. Analysis found: C 41.7, H 3.4, N 5.3%; C_9_H_9_NO_6_S requires: C 41.7, H 3.5, N 5.4%.

### Refinement

All H atoms were placed in idealized positions [C—H = 0.96 (methyl) and 0.93 Å(aromatic)] and included in the refinement in the riding-model approximation, with *U*_iso_(H)= 1.5 *U*_eq_(methyl C) and 1.2 *U*_eq_(aromatic C).

### Crystal data

**Table:**

C_9_H_9_NO_6_S	*F*(000) = 536
*M**_r_* = 259.23	*D*_x_ = 1.524 Mg m^−^^3^
Monoclinic, *P*2_1_/*c*	Mo *K*α radiation, λ = 0.71073 Å
Hall symbol: -P 2ybc	Cell parameters from 2311 reflections
*a* = 9.0108 (12) Å	θ = 2.9–25.0°
*b* = 8.7671 (11) Å	µ = 0.30 mm^−^^1^
*c* = 14.4761 (19) Å	*T* = 273 K
β = 98.955 (2)°	Block, colorless
*V* = 1129.7 (3) Å^3^	0.20 × 0.20 × 0.18 mm
*Z* = 4	

### Data collection

**Table:**

Bruker SMART APEX CCD detector diffractometer	2783 independent reflections
Radiation source: fine-focus sealed tube	2042 reflections with *I* > 2σ(*I*)
graphite	*R*_int_ = 0.028
phi and ω scans	θ_max_ = 28.3°, θ_min_ = 2.7°
Absorption correction: multi-scan (*SADABS*; Sheldrick, 1996)	*h* = −11→11
*T*_min_ = 0.942, *T*_max_ = 0.948	*k* = −11→10
9661 measured reflections	*l* = −19→19

### Refinement

**Table:**

Refinement on *F*^2^	Primary atom site location: structure-invariant direct methods
Least-squares matrix: full	Secondary atom site location: difference Fourier map
*R*[*F*^2^ > 2σ(*F*^2^)] = 0.041	Hydrogen site location: inferred from neighbouring sites
*wR*(*F*^2^) = 0.111	H-atom parameters constrained
*S* = 1.05	*w* = 1/[σ^2^(*F*_o_^2^) + (0.047*P*)^2^ + 0.3279*P*] where *P* = (*F*_o_^2^ + 2*F*_c_^2^)/3
2783 reflections	(Δ/σ)_max_ < 0.001
156 parameters	Δρ_max_ = 0.22 e Å^−^^3^
0 restraints	Δρ_min_ = −0.32 e Å^−^^3^

### Special details

**Table:**

Geometry. All e.s.d.'s (except the e.s.d. in the dihedral angle between two l.s. planes) are estimated using the full covariance matrix. The cell e.s.d.'s are taken into account individually in the estimation of e.s.d.'s in distances, angles and torsion angles; correlations between e.s.d.'s in cell parameters are only used when they are defined by crystal symmetry. An approximate (isotropic) treatment of cell e.s.d.'s is used for estimating e.s.d.'s involving l.s. planes.
Refinement. Refinement of *F*^2^ against ALL reflections. The weighted *R*-factor *wR* and goodness of fit *S* are based on *F*^2^, conventional *R*-factors *R* are based on *F*, with *F* set to zero for negative *F*^2^. The threshold expression of *F*^2^ > σ(*F*^2^) is used only for calculating *R*-factors(gt) *etc*. and is not relevant to the choice of reflections for refinement. *R*-factors based on *F*^2^ are statistically about twice as large as those based on *F*, and *R*- factors based on ALL data will be even larger.

### Fractional atomic coordinates and isotropic or equivalent isotropic displacement parameters (Å^2^)

**Table:**

	*x*	*y*	*z*	*U*_iso_*/*U*_eq_	
S1	−0.24972 (5)	0.42896 (5)	0.03839 (4)	0.04280 (16)	
O6	0.42147 (14)	0.11875 (17)	0.20037 (9)	0.0491 (4)	
O5	0.28877 (18)	−0.09358 (17)	0.15947 (13)	0.0665 (5)	
O4	0.32570 (17)	0.07267 (19)	−0.01616 (11)	0.0608 (4)	
O3	0.12224 (18)	0.08499 (19)	−0.11609 (9)	0.0581 (4)	
O2	−0.26056 (19)	0.53991 (19)	0.10923 (12)	0.0694 (5)	
O1	−0.24582 (17)	0.48263 (19)	−0.05419 (11)	0.0613 (4)	
N1	0.19276 (18)	0.10350 (18)	−0.03781 (11)	0.0399 (4)	
C1	−0.3960 (2)	0.2976 (3)	0.0338 (2)	0.0737 (8)	
H1A	−0.4905	0.3496	0.0188	0.111*	
H1B	−0.3909	0.2482	0.0934	0.111*	
H1C	−0.3872	0.2227	−0.0134	0.111*	
C2	−0.0851 (2)	0.3192 (2)	0.07342 (12)	0.0383 (4)	
C4	0.11351 (19)	0.16778 (19)	0.03452 (11)	0.0333 (4)	
C3	−0.01499 (19)	0.2509 (2)	0.00566 (12)	0.0361 (4)	
H3	−0.0535	0.2607	−0.0575	0.043*	
C5	0.1726 (2)	0.1479 (2)	0.12854 (12)	0.0380 (4)	
C8	0.3013 (2)	0.0425 (2)	0.16253 (13)	0.0417 (4)	
C7	−0.0276 (2)	0.3049 (3)	0.16756 (13)	0.0502 (5)	
H7	−0.0750	0.3526	0.2124	0.060*	
C6	0.1005 (2)	0.2193 (2)	0.19439 (13)	0.0489 (5)	
H6	0.1388	0.2094	0.2576	0.059*	
C9	0.5498 (2)	0.0281 (3)	0.24167 (17)	0.0684 (7)	
H9A	0.5205	−0.0387	0.2882	0.103*	
H9B	0.6285	0.0945	0.2703	0.103*	
H9C	0.5853	−0.0313	0.1939	0.103*	

### Atomic displacement parameters (Å^2^)

**Table:**

	*U*^11^	*U*^22^	*U*^33^	*U*^12^	*U*^13^	*U*^23^
S1	0.0366 (3)	0.0341 (3)	0.0543 (3)	0.00290 (19)	−0.0033 (2)	0.0009 (2)
O6	0.0375 (7)	0.0557 (9)	0.0497 (8)	−0.0009 (6)	−0.0073 (6)	0.0037 (6)
O5	0.0590 (10)	0.0406 (9)	0.0924 (12)	0.0075 (7)	−0.0115 (9)	0.0010 (8)
O4	0.0422 (8)	0.0762 (11)	0.0641 (10)	0.0123 (8)	0.0087 (7)	−0.0088 (8)
O3	0.0607 (9)	0.0771 (11)	0.0362 (8)	0.0002 (8)	0.0061 (7)	−0.0069 (7)
O2	0.0670 (11)	0.0601 (10)	0.0752 (11)	0.0246 (8)	−0.0075 (8)	−0.0210 (8)
O1	0.0565 (10)	0.0612 (10)	0.0608 (9)	0.0055 (8)	−0.0081 (7)	0.0182 (8)
N1	0.0409 (9)	0.0378 (9)	0.0414 (9)	−0.0030 (7)	0.0072 (7)	0.0010 (6)
C1	0.0395 (12)	0.0476 (14)	0.134 (2)	−0.0002 (10)	0.0131 (13)	0.0101 (14)
C2	0.0344 (9)	0.0383 (10)	0.0400 (10)	0.0025 (8)	−0.0006 (7)	0.0024 (7)
C4	0.0332 (9)	0.0318 (9)	0.0341 (9)	−0.0039 (7)	0.0029 (7)	−0.0008 (7)
C3	0.0353 (9)	0.0368 (10)	0.0341 (9)	−0.0032 (7)	−0.0010 (7)	0.0025 (7)
C5	0.0376 (9)	0.0357 (10)	0.0380 (9)	0.0016 (7)	−0.0022 (7)	−0.0004 (7)
C8	0.0387 (10)	0.0458 (12)	0.0383 (10)	0.0032 (8)	−0.0015 (7)	0.0010 (8)
C7	0.0546 (12)	0.0569 (13)	0.0381 (10)	0.0170 (10)	0.0038 (9)	−0.0044 (9)
C6	0.0561 (12)	0.0555 (12)	0.0318 (9)	0.0145 (10)	−0.0037 (8)	−0.0028 (9)
C9	0.0412 (12)	0.0959 (19)	0.0622 (14)	0.0130 (12)	−0.0100 (10)	0.0143 (13)

### Geometric parameters (Å, °)

**Table:**

S1—O1	1.4260 (16)	C2—C3	1.383 (3)
S1—O2	1.4278 (16)	C2—C7	1.386 (2)
S1—C1	1.744 (2)	C4—C3	1.377 (2)
S1—C2	1.7749 (18)	C4—C5	1.393 (2)
O6—C8	1.317 (2)	C3—H3	0.9300
O6—C9	1.453 (2)	C5—C6	1.384 (3)
O5—C8	1.198 (2)	C5—C8	1.504 (3)
O4—N1	1.220 (2)	C7—C6	1.381 (3)
O3—N1	1.221 (2)	C7—H7	0.9300
N1—C4	1.469 (2)	C6—H6	0.9300
C1—H1A	0.9600	C9—H9A	0.9600
C1—H1B	0.9600	C9—H9B	0.9600
C1—H1C	0.9600	C9—H9C	0.9600
			
O1—S1—O2	117.69 (11)	C4—C3—C2	117.99 (15)
O1—S1—C1	108.11 (12)	C4—C3—H3	121.0
O2—S1—C1	109.99 (13)	C2—C3—H3	121.0
O1—S1—C2	107.80 (9)	C6—C5—C4	117.83 (16)
O2—S1—C2	108.17 (9)	C6—C5—C8	118.24 (16)
C1—S1—C2	104.24 (10)	C4—C5—C8	123.69 (17)
C8—O6—C9	116.36 (18)	O5—C8—O6	125.95 (18)
O4—N1—O3	123.86 (17)	O5—C8—C5	122.42 (17)
O4—N1—C4	117.96 (15)	O6—C8—C5	111.49 (17)
O3—N1—C4	118.18 (15)	C6—C7—C2	119.59 (18)
S1—C1—H1A	109.5	C6—C7—H7	120.2
S1—C1—H1B	109.5	C2—C7—H7	120.2
H1A—C1—H1B	109.5	C7—C6—C5	120.91 (17)
S1—C1—H1C	109.5	C7—C6—H6	119.5
H1A—C1—H1C	109.5	C5—C6—H6	119.5
H1B—C1—H1C	109.5	O6—C9—H9A	109.5
C3—C2—C7	121.07 (17)	O6—C9—H9B	109.5
C3—C2—S1	119.07 (13)	H9A—C9—H9B	109.5
C7—C2—S1	119.85 (15)	O6—C9—H9C	109.5
C3—C4—C5	122.57 (16)	H9A—C9—H9C	109.5
C3—C4—N1	117.78 (15)	H9B—C9—H9C	109.5
C5—C4—N1	119.60 (15)		
			
O1—S1—C2—C3	−25.21 (18)	N1—C4—C5—C6	175.52 (17)
O2—S1—C2—C3	−153.45 (16)	C3—C4—C5—C8	172.23 (17)
C1—S1—C2—C3	89.52 (18)	N1—C4—C5—C8	−10.2 (3)
O1—S1—C2—C7	153.91 (17)	C9—O6—C8—O5	0.2 (3)
O2—S1—C2—C7	25.7 (2)	C9—O6—C8—C5	175.83 (17)
C1—S1—C2—C7	−91.4 (2)	C6—C5—C8—O5	103.2 (2)
O4—N1—C4—C3	157.53 (17)	C4—C5—C8—O5	−71.0 (3)
O3—N1—C4—C3	−22.0 (2)	C6—C5—C8—O6	−72.6 (2)
O4—N1—C4—C5	−20.2 (2)	C4—C5—C8—O6	113.1 (2)
O3—N1—C4—C5	160.31 (17)	C3—C2—C7—C6	−0.9 (3)
C5—C4—C3—C2	1.4 (3)	S1—C2—C7—C6	179.96 (17)
N1—C4—C3—C2	−176.25 (15)	C2—C7—C6—C5	0.2 (3)
C7—C2—C3—C4	0.2 (3)	C4—C5—C6—C7	1.2 (3)
S1—C2—C3—C4	179.26 (13)	C8—C5—C6—C7	−173.4 (2)
C3—C4—C5—C6	−2.1 (3)		

### Hydrogen-bond geometry (Å, °)

**Table:**

*D*—H···*A*	*D*—H	H···*A*	*D*···*A*	*D*—H···*A*
C6—H6···O2^i^	0.93	2.54	3.370 (2)	148
C6—H6···O3^ii^	0.93	2.59	3.216 (2)	125

Symmetry codes: (i) −*x*, *y*−1/2, −*z*+1/2; (ii) *x*, −*y*+1/2, *z*+1/2.
